# Supplementary material for: Regulated repression governs the cell fate promoter controlling yeast meiosis
Source: Nat Commun. 2020 May 8;11:2271. doi: 10.1038/s41467-020-16107-w (PMC7210989; doi:10.1038/s41467-020-16107-w)
Supplement: Supplementary file 1 — Supplementary Information [file 41467_2020_16107_MOESM1_ESM.pdf]

Supplementary Information

Regulated repression governs the cell fate promoter controlling yeast meiosis

Tam et *al*.

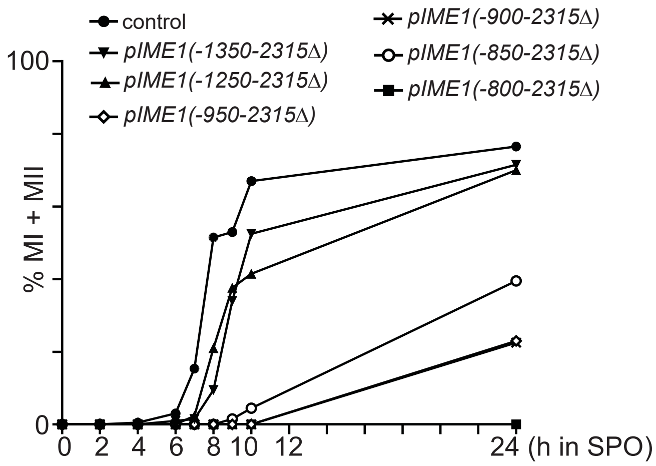

Supplementary Fig. 1. The effect of truncations in the *IME1* promoter on meiosis.

The effect of truncations in the *IME1* promoter on meiosis. For the analyses we used diploid cells harbouring one copy of *IME1* deleted (control, FW4128), while different promoter deletion mutants were generated at the wild-type *IME1* locus (*pIME1(-1350-2315Δ)*, FW4781; *pIME1(-1250-2315Δ)*, FW4780; *pIME1(-950-2315Δ)*, FW4779; *pIME1(-900-2315Δ)*, FW4778; *pIME1(-850-2315Δ)*, FW4777; *pIME1(-800-2315Δ)*, FW3944).
Control and mutant cells were grown to saturation in rich medium (YPD), grown for an additional 16 to 18 hours in pre-sporulation medium (BYTA), and subsequently cells were shifted to sporulation medium (SPO). Samples were taken at the indicated time points, fixed, and DAPI masses were counted cells per sample) to determine the percentage of cells that underwent meiosis (MI+MII). Cells harbouring two, three, or four DAPI masses were classified as meiosis. At least 190 cells were analysed per sample.

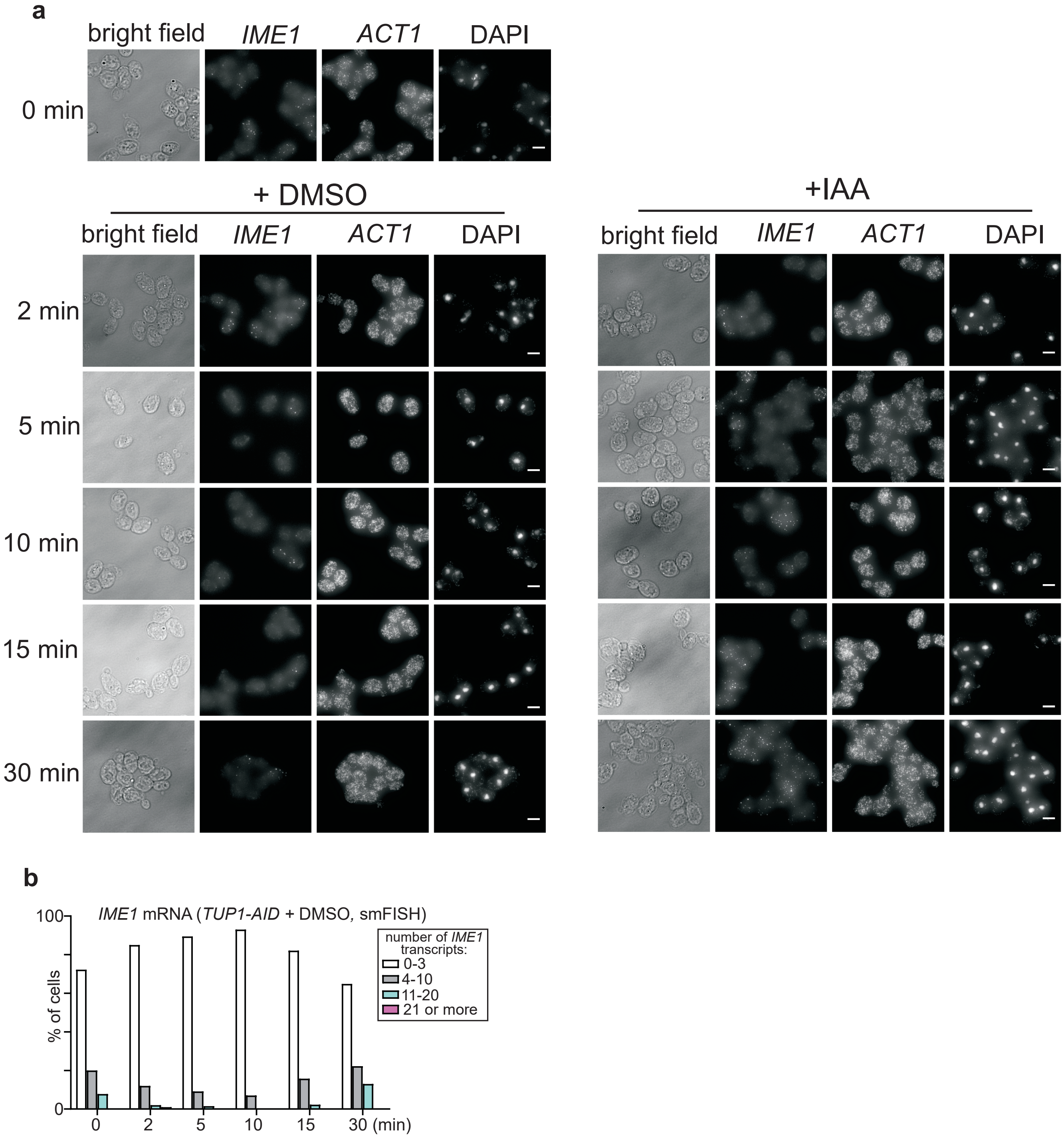

Supplementary Fig. 2. Single cell analysis of *IME1* expression upon Tup1 depletion.

a Representative images of smFISH data described in Fig. 1h. Cells were fixed, and hybridized with *IME1* (AF594) and *ACT1* (Cy5) probes. Representative images (n≥50 cells) representing bright field, *IME1*, *ACT1*, and DAPI are displayed. Scale bar represents 5 µm.

b Same data as described in Fig. 1h, except that the single cell data for *IME1* expression in *TUP1-AID*+DMSO were binned according to expression levels.

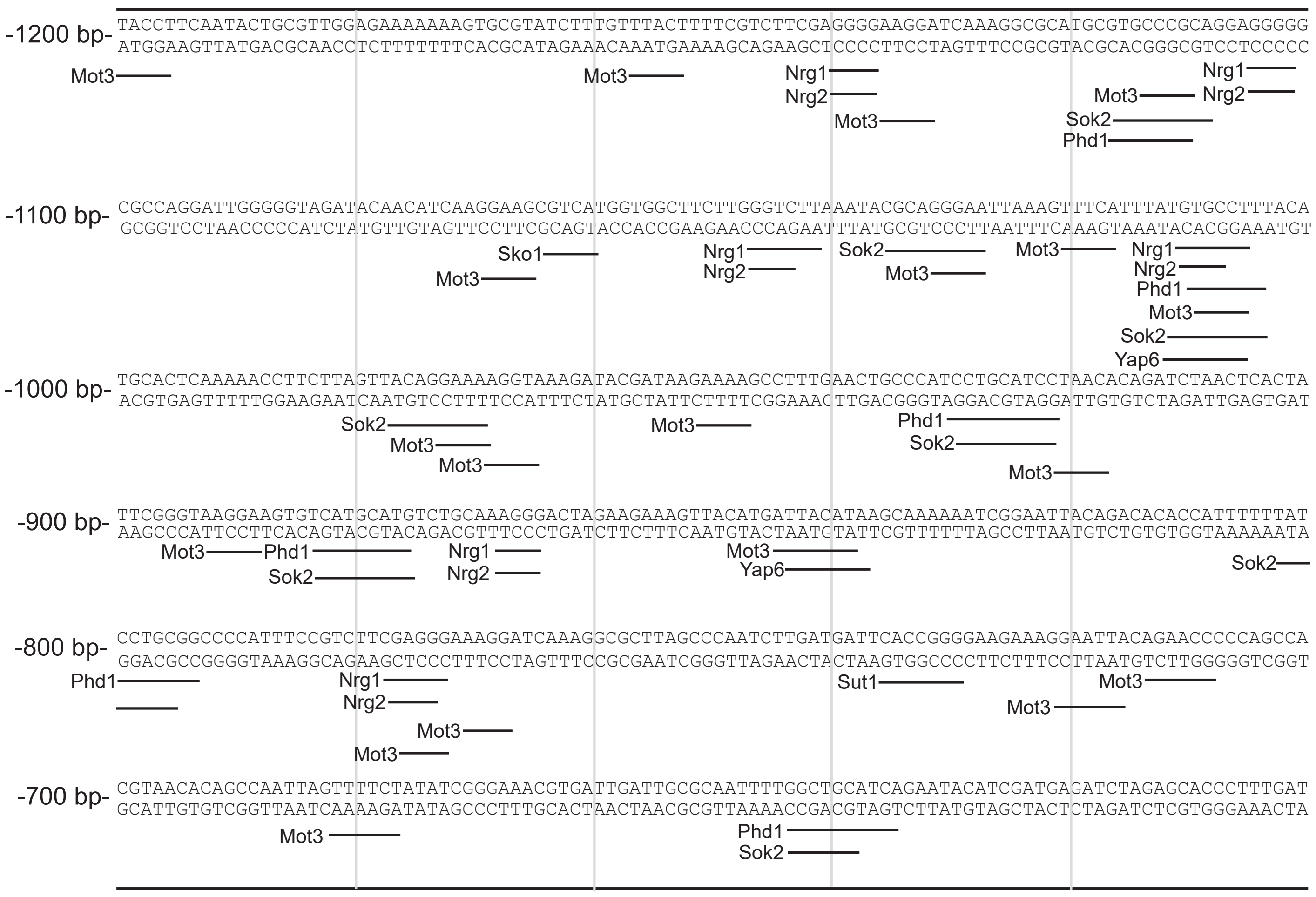

Supplementary Fig. 3. TF binding motifs in the *IME1* promoter.

Sequence of the *IME1* promoter spanning the region between 600 and 1200 bp upstream of the AUG start codon of *IME1*. Highlighted are the transcription factor binding motifs in the *IME1* promoter.

a

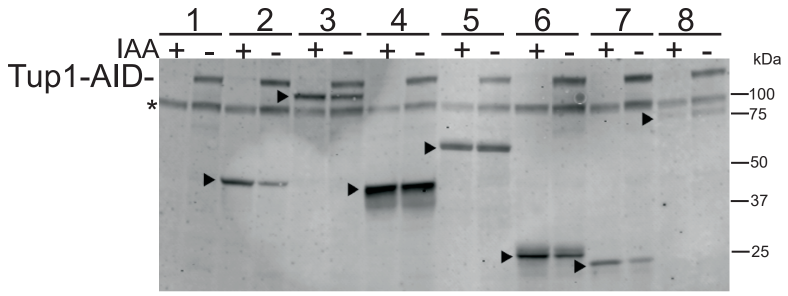

1. Tup1-AID
2. Tup1-AID + Yap6-V5
3. Tup1-AID + Sok2-V5
4. Tup1-AID + Phd1-V5
5. Tup1-AID + Mot3-V5
6. Tup1-AID + Nrg1-V5
7. Tup1-AID + Nrg2-V5
8. Tup1-AID + Sko1-V5

b

Putative BS in -700 to -1100		Interacts with Tup1-Cyc8
Yap6	2x	y
Sok2	6x	nd
Phd1	4x	y
Nrg1	4x	y
Nrg2	4x	y
Sut1	1x	y
Mot3	14x	y
Sko1	1x	y

c

*pIME1-bs*Δ

```
-1100 bp- CGCCAGGATTGGGGGTAGATACAACATCAAttcAGatgacTGGTGGCTTCTTttttTCTTAAATACtactttcATTAAAGggTgATTTAgtgtaagTTcac
          GCGGTCCTAACCCCCATCTATGTTGTAGTTaagTCtactgACCACCGAAGAAaaaAGAATTTATGatgaaagTAATTTCccAcTAAATcacattcAAgtg

-1000 bp- gtacCTCAAAAACCTTCTTAGTTACcttccActtTcAAGATACGATAAtAccAGCCTTTGAACTGCCCATCagtacgaaTAACACAGATCTAACTCACTA
          catgGAGTTTTTGGAAGAATCAATGgaaggTgaaAgTTCTATGCTATTaTggTCGGAAACTTGACGGGTAGtcatgcttATTGTGTCTAGATTGAGTGAT

-900 bp-  TTCGGGTAAttcAGTGTCAgtacTGTagtaAActttACTAGAAGAAAGTTACATGAggcacgccGCAAAAAATCGGAATTACAGACACACCATTTTTTAT
          AAGCCCATTaagTCACAGTcatgACAtcatTTgaaaTGATCTTCTTTCAATGTACTccgtgcggCGTTTTTTAGCCTTAATGTCTGTGTGGTAAAAAATA

-800 bp-  aagtaGtaCCCATTTCCGTCTTCGctttcAAttcTCAAAGGCGCTTAGCCCAATCTTGATGATTCACCGGGGAAGAAAttcATgcaAtAcaaCCCAGCCA
          ttcatCatGGGTAAAGGCAGAAGCgaaagTTaagAGTTTCCGCGAATCGGGTTAGAACTACTAAGTGGCCCCTTCTTTaagTAcgtTaTgttGGGTCGGT
```

Supplementary Fig. 4. Expression levels of candidate TFs, number TF binding motifs, and the sequence of the *pIME1-bs*Δ construct.

a Western blot analysis of samples described in Fig. 3b. Diploid cells harbouring *TUP1-AID* and V5-tagged TFs (*YAP6-V5,* FW4214; *SOK2-V5*, FW4218; *PHD1-V5*, FW5056; *MOT3-V5*, FW4229; *NRG1-V5*, FW4230; *NRG2-V5*, FW5055; *SKO1-V5*, FW4224) were grown to exponential phase. As a control *TUP1-AID* cells (FW5057) were included, which also harbour a V5 tag. Cells were either treated with IAA (+) or DMSO (-), and the expression for each transcription factor and Tup1-AID was determined by western blot using anti-V5 antibodies. Highlighted are the bands representing the different TFs. A representative AID depletion experiment is shown (n>3).

b Table describing the sequence motifs identified in the *IME1* promoter. Data are shown for TFs that associate with the *IME1* promoter as shown in Fig. 2b, and for the region between 700 and 1100 bp upstream of *IME1* AUG. The number of binding sites as well as whether the transcription factor is known to interact with Tup1-Cyc8 are displayed. BS = binding site.

c Sequence displaying the mutated sites (in red lowercase) in the *IME1* promoter of the *pIME1-bs*Δ construct.

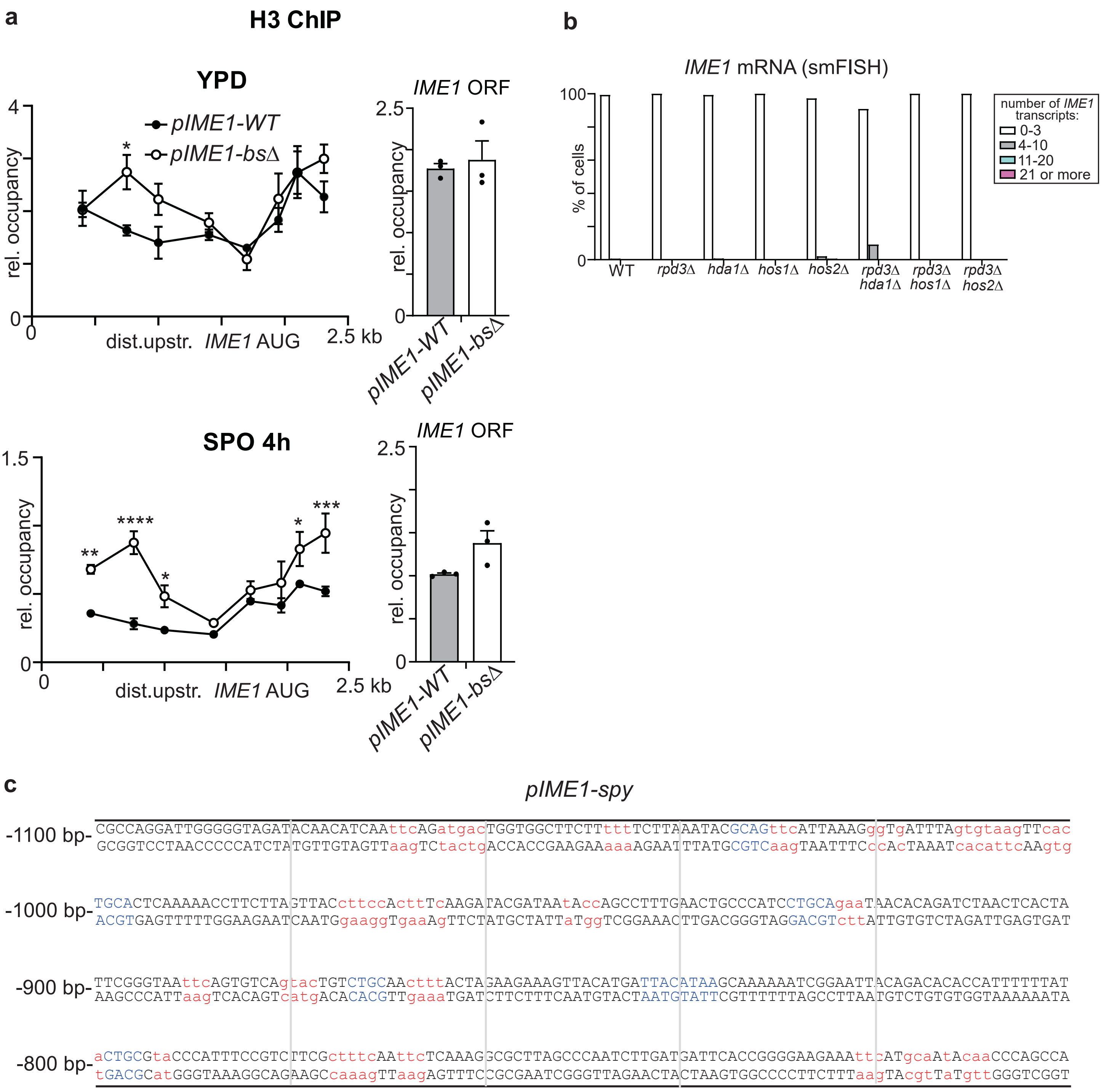

Supplementary Fig. 5. Analysis of chromatin state, HDACs, and the sequence of *pIME1-spy* construct.
a Histone H3 occupancy as determined by ChIP across the *IME1* promoter and *IME1* ORF in *pIME1-WT* (FW5370) and *pIME1-bsΔ* cells (FW5372) in rich nutrient conditions (YPD) and during entry into meiosis (SPO 4h). Signals were normalized over *HMR*. The mean and SEM of n=3 are shown. Two-way ANOVA analysis was carried out using the uncorrected Fisher's LSD method with 95% confidence. *P*-values that are statistically significant (* = ≤ 0.05, ** = ≤ 0.01, *** = ≤ 0.001, **** = ≤ 0.0001) are indicated.
b Same data as in Fig. 4c, except that the single cell data for *IME1* were binned according to expression levels.
c Sequence displaying the wild-type motif sites restored (in blue uppercase) and the mutated sites (in red lowercase) in the *IME1* promoter of the *pIME1-spy* construct.

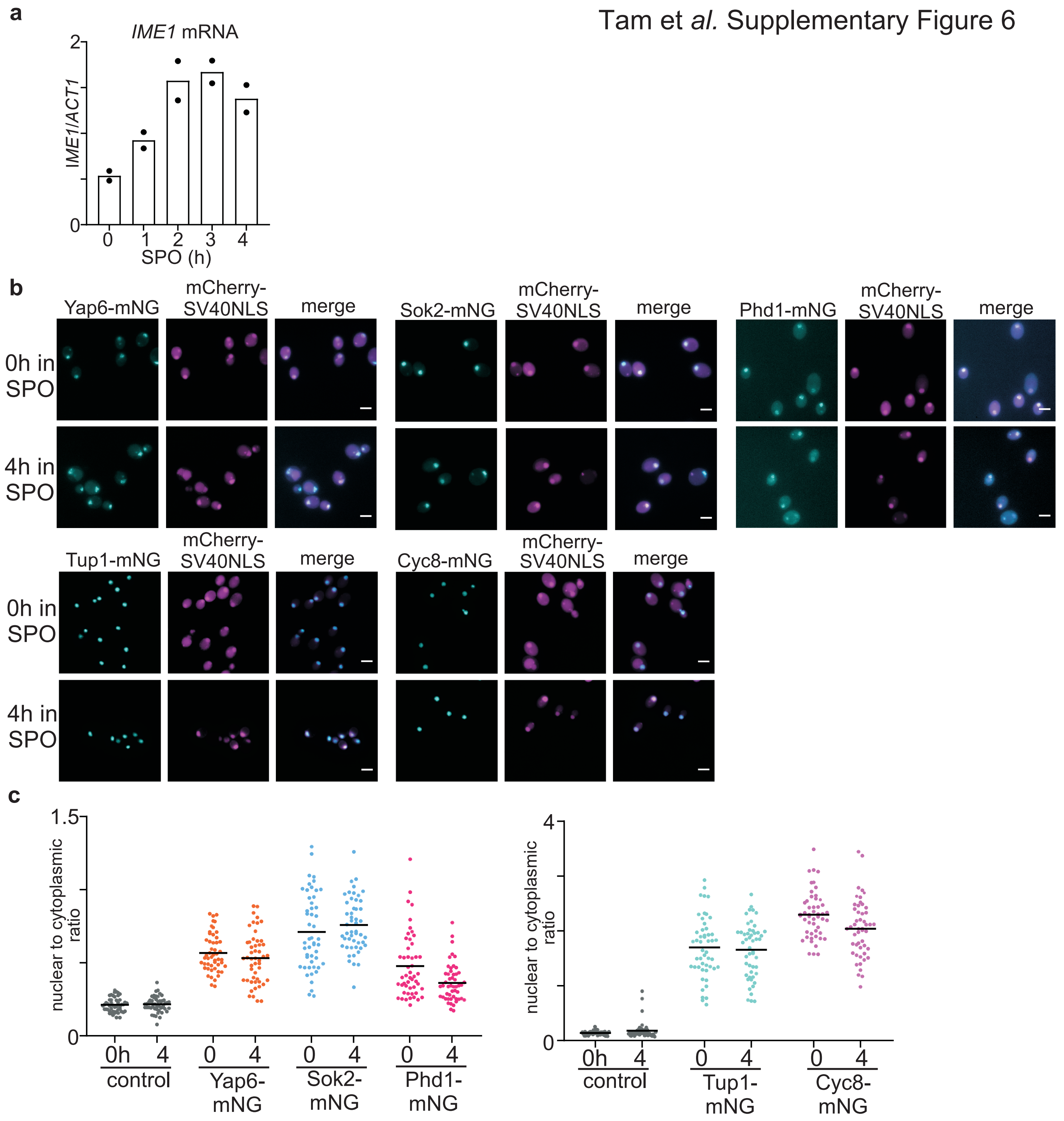

Supplementary Fig. 6. Expression and localization of Tup1-Cyc8 and TFs.

a *IME1* expression in wild-type cells (FW1511) during entry into meiosis as detected by RT-qPCR. Multiple time points were taken in SPO to analyse *IME1* expression levels during entry into meiosis. The signals were normalized over the *ACT1* gene. The mean value of n=2 is displayed.

b Representative images (n=50 cells per sample) of Yap6-mNG (FW7473), Sok2-mNG (FW7475), Phd1-mNG (FW7477), Tup1-mNG (FW7644), and Cyc8-mNG (FW7642) localization prior to (0 hours in SPO) and during entry into meiosis (4 hours in SPO). Each transcription factor was fused to mNeongreen (mNG). These cells also expressed mCherry fused to SV40 nuclear localization signal (NLS) (mCherry-NLS) to determine nuclear localization. Scale bar represents 5µm.

c Nuclear to cytoplasmic ratio of the mNG-tagged TFs described in b. As a control, the signals of cells harbouring no mNG-tag (FW5199) are displayed. The black bar indicates the mean signal, and each point displays a single cell measurement.

Tam et *al.* Supplementary Figure 7

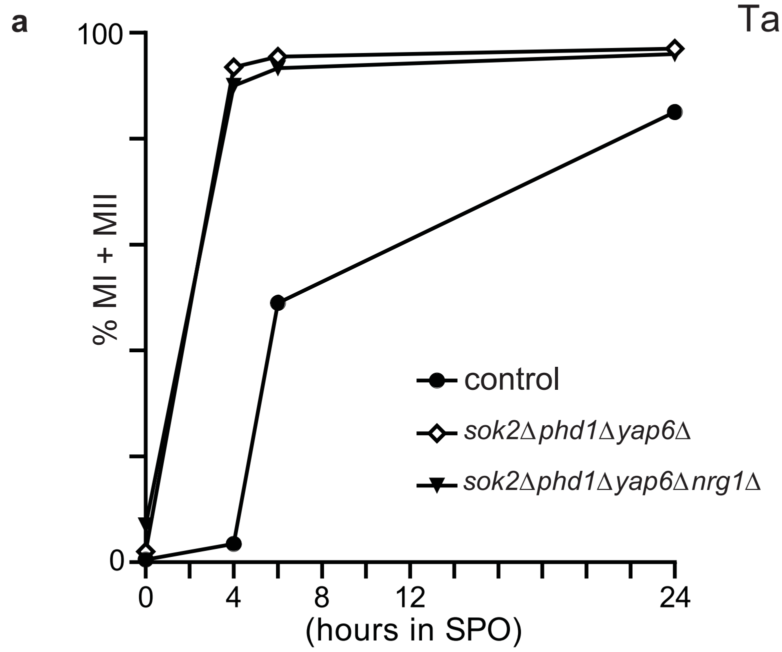

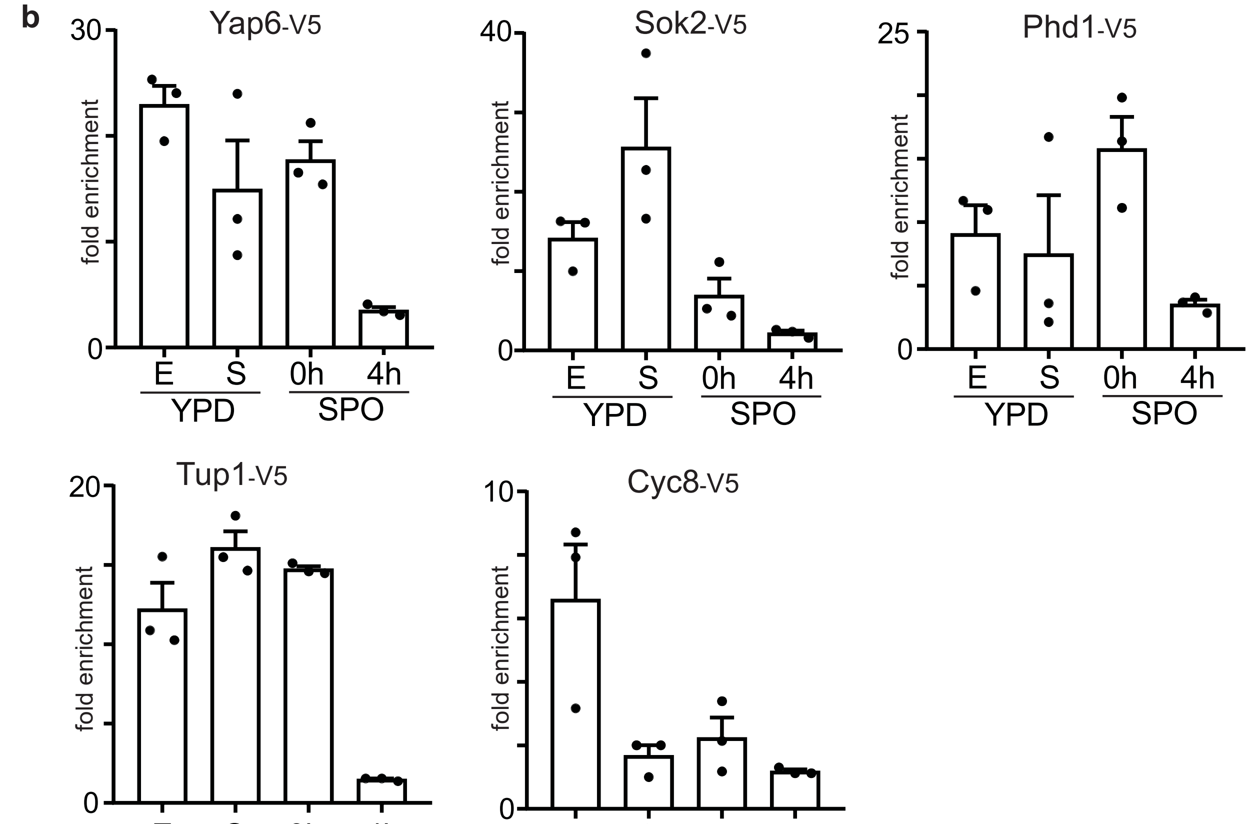

Supplementary Fig. 7. The effect of TF deletion mutants on meiosis and, binding of TFs and Tup1-Cyc8 under different growth conditions.

a Meiosis in wild-type (FW3456), *sok2Δphd1Δyap6Δ* triple mutant (FW4010), and *sok2Δphd1Δyap6Δnrg1Δ* quadruple mutant cells (FW5657). Cells were induced to enter meiosis in SPO. DAPI masses were counted for the indicated time points to determine the percentage of cells that underwent meiosis (MI+MII). Cells harbouring two, three, or four DAPI masses were classified as meiosis. At least 190 cells were analysed.

b Binding of Yap6, Sok2, Phd1, Tup1, and Cyc8 to the *IME1* promoter under different nutrient conditions as determined by ChIP. Cells harbouring V5 epitope tagged version of each transcription factor (Yap6-V5, FW3833; Sok2-V5, FW5638; Phd1-V5, FW4466; Tup1-V5, FW3456; Cyc8-V5, FW6381) were grown till exponential growth (E) or saturation (S) in YPD, or grown for an additional 16 to 18 hours in pre-sporulation medium (0h) and shifted to sporulation medium for four hours (4h). ChIP signals were normalized over *HMR*. The mean and SEM of n=3 are displayed.

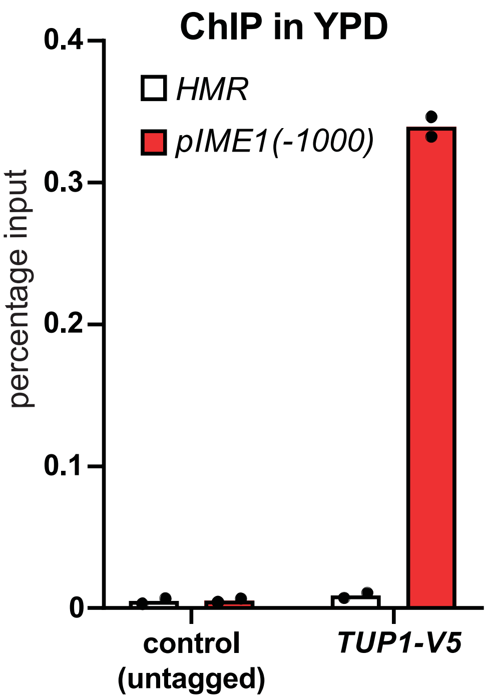

Supplementary Fig. 8. Tup1 does not associate with the region of the *HMR* locus used as negative control in ChIP experiments througout this manuscript.

Tup1 enrichment at the *HMR* locus and *IME1* promoter as determined by ChIP, normalized over input. Diploid cells harbouring V5 epitope-tagged Tup1 (FW3456) were grown in rich medium (YPD) to the exponential phase. As a control, wild-type cells (untagged, FW1511) were included in the analysis. The region around 1000 bp upstream of the AUG of *IME1* (*pIME1(-1000)*) and the *HMR* locus were analysed for Tup1-V5 enrichment. ChIP signals are presented as a percentage over input. The mean signals of n=2 are displayed.

Supplementary Table 1. Oligo nucleotide sequences used throughout this study

Primer	Oligo sequence (5' to 3')	Targeted region
oFW43	acgatccccgtccaagttatg	*HMR1* (forward)
oFW50	cttcaaaggagtcttaatttccctg	*HMR1* (reverse)
oFW106	gtaccaccatgttcccaggtatt	*ACT1* (forward)
oFW268	agatggaccactttcgtcgt	*ACT1* (reverse)
oFW493	gatggagggttggcataaaa	2310 bp upstream of *IME1* (forward)
oFW494	tgacggtgacgtacgatctcta	2310 bp upstream of *IME1* (reverse)
oFW248	ccgtatggtgttggagtaatttg	2100 bp upstream of *IME1* (forward)
oFW249	tgccatttagtggacttcttgag	2100 bp upstream of *IME1* (reverse)
oFW481	atttttagcgactgccgaaa	1950 bp upstream of *IME1* (forward)
oFW482	atgcaacgcctacttgtttt	1950 bp upstream of *IME1* (reverse)
oFW127	gccaacttggagaaagaatgtg	1700 bp upstream of *IME1* (forward)
oFW128	cggaggtactagtcatcggaat	1700 bp upstream of *IME1* (reverse)
oFW254	agaaacgcaaatgctcagagag	1400 bp upstream of *IME1* (forward)
oFW255	gaggtaatagcggatgacatcaa	1400 bp upstream of *IME1* (reverse)
oFW539	gggtcttaaatacgcagggaat	1000 bp upstream of *IME1* (forward)
oFW540	ggcagttcaaaggcttttctta	1000 bp upstream of *IME1* (reverse)
oFW2685	aggattgggggtagatacaacatc	1000 bp upstream of *IME1* (forward)<br>for *pIME1-WT*, *pIME1-bs*Δ, *pIME1-spy*
oFW2688	gatgggcagttcaaaggct	1000 bp upstream of *IME1* (reverse)<br>for *pIME1-WT*, *pIME1-bs*Δ, *pIME1-spy*
oFW333	cttcgagggaaaggatcaaag	750 bp upstream of *IME1* (forward)
oFW334	ggctgggggttctgtaattc	750 bp upstream of *IME1* (reverse)
oFW161	taaacaacaacaacaacgcaca	400 bp upstream of *IME1* (forward)
oFW162	ggcaaggaacaagatcaaaaac	400 bp upstream of *IME1* (reverse)
oFW463	caacgcctccgataatgtatatg	*IME1* (forward)
oFW464	acgtcgaaggcaatttctaatg	*IME1* (reverse)
