# Supplementary material for: Regulated repression governs the cell fate promoter controlling yeast meiosis
Source: Nat Commun. 2020 May 8;11:2271. doi: 10.1038/s41467-020-16107-w (PMC7210989; doi:10.1038/s41467-020-16107-w)
Supplement: Supplementary file 3 — Description of Additional Supplementary Files [file 41467_2020_16107_MOESM3_ESM.pdf]

Description of Additional Supplementary Files

File Name: Supplementary Data 1
Description: Genotypes of strains used throughout this study.
