# Supplementary material for: Regulated repression governs the cell fate promoter controlling yeast meiosis
Source: Nat Commun. 2020 May 8;11:2271. doi: 10.1038/s41467-020-16107-w (PMC7210989; doi:10.1038/s41467-020-16107-w)
Supplement: Supplementary file 4 — Supplementary Data 1 [file 41467_2020_16107_MOESM4_ESM.docx]

| **Supplementary Data 1. Genotypes of strains used throughout this study** | |
| --- | --- |
| **Yeast strain** | **Genotype** |
| FW1511 (*WT*) | *MATa, ho::LYS2, lys2, ura3, leu2::hisG, his3::hisG, trp1::hisG MATalpha, ho::LYS2, lys2, ura3, leu2::hisG, his3::hisG, trp1::hisG* |
| FW4128 | *MATa, ho::LYS2, lys2, ura3, leu2::hisG, his3::hisG, trp1::hisG MATalpha, ho::LYS2, lys2, ura3, leu2::hisG, his3::hisG, trp1::hisG, ime1::HISMX6* |
| FW3946 | *MATa, ho::LYS2, lys2, ura3, leu2::hisG, his3::hisG, trp1::hisG, pIME1(-1600-2315)::KanMX MATalpha, ho::LYS2, lys2, ura3, leu2::hisG, his3::hisG, trp1::hisG, ime1::HISMX6* |
| FW3947 | *MATa, ho::LYS2, lys2, ura3, leu2::hisG, his3::hisG, trp1::hisG, pIME1(-1400-2315)::KanMX MATalpha, ho::LYS2, lys2, ura3, leu2::hisG, his3::hisG, trp1::hisG, ime1::HISMX6* |
| FW3948 | *MATa, ho::LYS2, lys2, ura3, leu2::hisG, his3::hisG, trp1::hisG, pIME1(-1200-2315)::KanMX MATalpha, ho::LYS2, lys2, ura3, leu2::hisG, his3::hisG, trp1::hisG, ime1::HISMX6* |
| FW3949 | *MATa, ho::LYS2, lys2, ura3, leu2::hisG, his3::hisG, trp1::hisG, pIME1(-1000-2315)::KanMX MATalpha, ho::LYS2, lys2, ura3, leu2::hisG, his3::hisG, trp1::hisG, ime1::HISMX6* |
| FW3950 | *MATa, ho::LYS2, lys2, ura3, leu2::hisG, his3::hisG, trp1::hisG, pIME1(-800-2315)::KanMX MATalpha, ho::LYS2, lys2, ura3, leu2::hisG, his3::hisG, trp1::hisG, ime1::HISMX6* |
| FW3951 | *MATa, ho::LYS2, lys2, ura3, leu2::hisG, his3::hisG, trp1::hisG, pIME1(-600-2315)::KanMX MATalpha, ho::LYS2, lys2, ura3, leu2::hisG, his3::hisG, trp1::hisG, ime1::HISMX6* |
| FW6381 | *MATa, ho::LYS2, lys2, ura3, leu2::hisG, his3::hisG, trp1::hisG, CYC8-3V5::KanMX MATalpha, ho::LYS2, lys2, ura3, leu2::hisG, his3::hisG, trp1::hisG, CYC8-3V5::KanMX* |
| FW968 | *MATa, ho::LYS2, ura3, leu2::hisG, his3::hisG, trp1::hisG*, *POG1-3xV5::HIS3 MATalpha, ho::LYS2, ura3, leu2::hisG, his3::hisG, trp1::hisG*, *POG1-3xV5::HIS3* |
| FW3944 | *MATa, ho::LYS2, lys2, ura3, leu2::hisG, his3::hisG, trp1::hisG, pIME1(-800-2315)::HIS3MX MATalpha, ho::LYS2, lys2, ura3, leu2::hisG, his3::hisG, trp1::hisG, ime1::HISMX6* |
| FW4777 | *MATa, ho::LYS2, lys2, ura3, leu2::hisG, his3::hisG, trp1::hisG, pIME1(-850-2315)::HIS3MX MATalpha, ho::LYS2, lys2, ura3, leu2::hisG, his3::hisG, trp1::hisG, ime1::HISMX6* |
| FW4778 | *MATa, ho::LYS2, lys2, ura3, leu2::hisG, his3::hisG, trp1::hisG, pIME1(-900-2315)::HIS3MX MATalpha, ho::LYS2, lys2, ura3, leu2::hisG, his3::hisG, trp1::hisG, ime1::HISMX6* |
| FW4779 | *MATa, ho::LYS2, lys2, ura3, leu2::hisG, his3::hisG, trp1::hisG, pIME1(-950-2315)::HIS3MX MATalpha, ho::LYS2, lys2, ura3, leu2::hisG, his3::hisG, trp1::hisG, ime1::HISMX6* |
| FW4780 | *MATa, ho::LYS2, lys2, ura3, leu2::hisG, his3::hisG, trp1::hisG, pIME1(-1250-2315)::HIS3MX MATalpha, ho::LYS2, lys2, ura3, leu2::hisG, his3::hisG, trp1::hisG, ime1::HISMX6* |
| FW4781 | *MATa, ho::LYS2, lys2, ura3, leu2::hisG, his3::hisG, trp1::hisG, pIME1(-1350-2315)::HIS3MX MATalpha, ho::LYS2, lys2, ura3, leu2::hisG, his3::hisG, trp1::hisG, ime1::HISMX6* |
| FW5057 | *MATa, ho::LYS2, lys2, ura3, leu2::hisG, trp1::hisG, his3::pTEF1-osTIR::HIS3,*  *TUP1-3V5-IAA7::KanMX6 MATalpha, ho::LYS2, ura3, leu2::hisG, his3::hisG, trp1::hisG, his3::pTEF1-osTIR::HIS3,*  *TUP1-3V5-IAA7::KanMX6* |
| FW6371 | *MATa, ho::LYS2, lys2, ura3, leu2::hisG, his3::hisG, trp1::hisG, his3::pTEF1-osTIR::HIS3 ,*  *CYC8-3V5-IAA7::KanMX6 MATalpha, ho::LYS2, lys2, ura3, leu2::hisG, his3::hisG, trp1::hisG, his3::pTEF1-osTIR::HIS3,*  *CYC8-3V5-IAA7::KanMX6* |
| FW3833 | *MATa, ho::LYS2, lys2, ura3, leu2::hisG, his3::hisG, trp1::hisG, YAP6-3V5::KanMX MATalpha, ho::LYS2, lys2, ura3, leu2::hisG, his3::hisG, trp1::hisG, YAP6-3V5::KanMX* |
| FW4466 | *MATa, ho::LYS2, lys2, ura3, leu2::hisG, his3::hisG, trp1::hisG, PHD1-3V5::KanMX MATalpha, ho::LYS2, lys2, ura3, leu2::hisG, his3::hisG, trp1::hisG, PHD1-3V5::KanMX* |
| FW4393 | *MATa, ho::LYS2, lys2, ura3, leu2::hisG, his3::hisG, trp1::hisG, NRG1-3V5::KanMX MATalpha, ho::LYS2, lys2, ura3, leu2::hisG, his3::hisG, trp1::hisG, NRG1-3V5::KanMX* |
| FW4396 | *MATa, ho::LYS2, lys2, ura3, leu2::hisG, his3::hisG, trp1::hisG, NRG2-3V5::KanMX MATalpha, ho::LYS2, lys2, ura3, leu2::hisG, his3::hisG, trp1::hisG, NRG2-3V5::KanMX* |
| FW7072 | *MATa, ho::LYS2, lys2, ura3, leu2::hisG, his3::hisG, trp1::hisG, CIN5-3V5::KanMX MATalpha, ho::LYS2, lys2, ura3, leu2::hisG, his3::hisG, trp1::hisG, CIN5-3V5::KanMX* |
| FW4665 | *MATa, ho::LYS2, lys2, ura3, leu2::hisG, his3::hisG, trp1::hisG, MIG1-3V5::KanMX MATalpha, ho::LYS2, lys2, ura3, leu2::hisG, his3::hisG, trp1::hisG, MIG1-3V5::KanMX* |
| FW6974 | *MATa, ho::LYS2, lys2, ura3, leu2::hisG, his3::hisG, trp1::hisG, SUT1-3V5::KanMX MATalpha, ho::LYS2, lys2, ura3, leu2::hisG, his3::hisG, trp1::hisG, SUT1-3V5::KanMX* |
| FW4383 | *MATa, ho::LYS2, lys2, ura3, leu2::hisG, his3::hisG, trp1::hisG, MOT3-3V5::KanMX MATalpha, ho::LYS2, lys2, ura3, leu2::hisG, his3::hisG, trp1::hisG, MOT3-3V5::KanMX* |
| FW4389 | *MATa, ho::LYS2, lys2, ura3, leu2::hisG, his3::hisG, trp1::hisG, SKO1-3V5::KanMX MATalpha, ho::LYS2, lys2, ura3, leu2::hisG, his3::hisG, trp1::hisG, SKO1-3V5::KanMX* |
| FW7070 | *MATa, ho::LYS2, lys2, ura3, leu2::hisG, his3::hisG, trp1::hisG, SFL1-3V5::KanMX MATalpha, ho::LYS2, lys2, ura3, leu2::hisG, his3::hisG, trp1::hisG, SFL1-3V5::KanMX* |
| FW4386 | *MATa, ho::LYS2, lys2, ura3, leu2::hisG, his3::hisG, trp1::hisG, RGT1-3V5::KanMX MATalpha, ho::LYS2, lys2, ura3, leu2::hisG, his3::hisG, trp1::hisG, RGT1-3V5::KanMX* |
| FW5638 | *MATa, ho::LYS2, ura3, leu2::hisG, his3::hisG, trp1::hisG, SOK2-3V5::HIS3 MATalpha, ho::LYS2, ura3, leu2::hisG, his3::hisG, trp1::hisG, SOK2-3V5::HIS3* |
| FW4399 | *MATa, ho::LYS2, lys2, ura3, leu2::hisG, his3::hisG, trp1::hisG, SKN7-3V5::KanMX MATalpha, ho::LYS2, lys2, ura3, leu2::hisG, his3::hisG, trp1::hisG, SKN7-3V5::KanMX* |
| FW4214 | *MATa, ho::LYS2, lys2, ura3, leu2::hisG, trp1::hisG, his3::pTEF1-osTIR::HIS3,*  *TUP1-3V5-IAA7::KanMX6, YAP6-3V5::KanMX MATalpha, ho::LYS2, lys2, ura3, leu2::hisG, trp1::hisG, his3::pTEF1-osTIR::HIS3,*  *TUP1-3V5-IAA7::KanMx6, YAP6-3V5::KanMX* |
| FW4218 | *MATa, ho::LYS2, lys2, ura3, leu2::hisG, trp1::hisG, his3::pTEF1-osTIR::HIS3,*  *TUP1-3V5-IAA7::KanMX6, SOK2-3V5::HIS3 MATalpha, ho::LYS2, lys2, ura3, leu2::hisG, trp1::hisG, his3::pTEF1-osTIR::HIS3,*  *TUP1-3V5-IAA7::KanMX6, SOK2-3V5::HIS3* |
| FW5056 | *MATa, ho::LYS2, lys2, ura3, leu2::hisG, trp1::hisG, his3::pTEF1-osTIR::HIS3,*  *TUP1-3V5-IAA7::KanMX6, PHD1-3V5::KanMX MATalpha, ho::LYS2, lys2, ura3, leu2::hisG, trp1::hisG, his3::pTEF1-osTIR::HIS3,*  *TUP1-3V5-IAA7::KanMX6, PHD1-3V5::KanMX* |
| FW4229 | *MATa, ho::LYS2, lys2, ura3, leu2::hisG, trp1::hisG, his3::pTEF1-osTIR::HIS3,*  *TUP1-3V5-IAA7::KanMX6, MOT3-3V5::KanMX MATalpha, ho::LYS2, lys2, ura3, leu2::hisG, trp1::hisG, his3::pTEF1-osTIR::HIS3,*  *TUP1-3V5-IAA7::KanMX6, MOT3-3V5::KanMX* |
| FW4230 | *MATa, ho::LYS2, lys2, ura3, leu2::hisG, trp1::hisG, his3::pTEF1-osTIR::HIS3,*  *TUP1-3V5-IAA7::KanMX6, NRG1-3V5::KanMX MATalpha, ho::LYS2, lys2, ura3, leu2::hisG, trp1::hisG, his3::pTEF1-osTIR::HIS3,*  *TUP1-3V5-IAA7::KanMX6, NRG1-3V5::KanMX* |
| FW5055 | *MATa, ho::LYS2, lys2, ura3, leu2::hisG, trp1::hisG, his3::pTEF1-osTIR::HIS3,*  *TUP1-3V5-IAA7::KanMX6, NRG2-3V5::KanMX MATalpha, ho::LYS2, lys2, ura3, leu2::hisG, trp1::hisG, his3::pTEF1-osTIR::HIS3,*  *TUP1-3V5-IAA7::KanMX6, NRG2-3V5::KanMX* |
| FW4224 | *MATa, ho::LYS2, lys2, ura3, leu2::hisG, trp1::hisG, his3::pTEF1-osTIR::HIS3,*  *TUP1-3V5-IAA7::KanMX6, SKO1-3V5::KanMX MATalpha, ho::LYS2, lys2, ura3, leu2::hisG, trp1::hisG, his3::pTEF1-osTIR::HIS3,*  *TUP1-3V5-IAA7::KanMX6, SKO1-3V5::KanMX* |
| FW3456 | *MATa, ho::LYS2, lys2, ura3, leu2::hisG, his3::hisG, trp1, TUP1-3V5::HIS3 MATalpha, ho::LYS2, lys2, ura3, leu2::hisG, his3::hisG, trp1, TUP1-3V5::HIS3* |
| FW5370 | *MATa, ho::LYS2, lys2, ura3, leu2::hisG, his3::hisG, trp1::hisG, pIME1-ime1::KanMX, trp1::pNH604-pIME1(WT)-sfGFP-IME1::TRP1, TUP1-3V5::HIS3 MATalpha, ho::LYS2, lys2, ura3, leu2::hisG, his3::hisG, trp1::hisG, pIME1-ime1::KanMX, trp1::pNH604-pIME1(WT)-sfGFP-IME1::TRP1, TUP1-3V5::HIS3* |
| FW5372 | *MATa, ho::LYS2, lys2, ura3, leu2::hisG, his3::hisG, trp1::hisG, pIME1-ime1::KanMX, trp1::pNH604-pIME1(bs∆)-sfGFP-IME1::TRP1, TUP1-3V5::HIS3 MATalpha, ho::LYS2, lys2, ura3, leu2::hisG, his3::hisG, trp1::hisG, pIME1-ime1::KanMX, trp1::pNH604-pIME1(bs∆)-sfGFP-IME1::TRP1, TUP1-3V5::HIS3* |
| FW8102 | *MATa, ho::LYS2, lys2, ura3, leu2::hisG, his3::hisG, trp1::hisG,*  *rpd3::HIS3MX, TUP1-3V5::HIS3 MATalpha, ho::LYS2, lys2, ura3, leu2::hisG, his3::hisG, trp1::hisG,*  *rpd3::HIS3MX, TUP1-3V5::HIS3* |
| FW8426 | *MATa, ho::LYS2, lys2, ura3, leu2::hisG, his3::hisG, trp1::hisG,*  *hda1::KanMX, TUP1-3V5::HIS3 MATalpha, ho::LYS2, lys2, ura3, leu2::hisG, his3::hisG, trp1::hisG,*  *hda1::KanMX, TUP1-3V5::HIS3* |
| FW8430 | *MATa, ho::LYS2, lys2, ura3, leu2::hisG, his3::hisG, trp1::hisG,*  *hos1::NatMX, TUP1-3V5::HIS3 MATalpha, ho::LYS2, lys2, ura3, leu2::hisG, his3::hisG, trp1::hisG,*  *hos1::NatMX, TUP1-3V5::HIS3* |
| FW8103 | *MATa, ho::LYS2, lys2, ura3, leu2::hisG, his3::hisG, trp1::hisG,*  *hos2:HIS3MX, TUP1-3V5::HIS3 MATalpha, ho::LYS2, lys2, ura3, leu2::hisG, his3::hisG, trp1::hisG,*  *hos2:HIS3MX, TUP1-3V5::HIS3* |
| FW8457 | *MATa, ho::LYS2, lys2, ura3, leu2::hisG, his3::hisG, trp1::hisG,*  *hda1::KanMX, rpd3::HIS3MX, TUP1-3V5::HIS3 MATalpha, ho::LYS2, lys2, ura3, leu2::hisG, his3::hisG, trp1::hisG,*  *hda1::KanMX, rpd3::HIS3MX, TUP1-3V5::HIS3* |
| FW8428 | *MATa, ho::LYS2, lys2, ura3, leu2::hisG, his3::hisG, trp1::hisG,*  *hos1::NatMX, rpd3::HIS3MX, TUP1-3V5::HIS3 MATalpha, ho::LYS2, lys2, ura3, leu2::hisG, his3::hisG, trp1::hisG,*  *hos1::NatMX, rpd3::HIS3MX, TUP1-3V5::HIS3* |
| FW8171 | *MATa, ho::LYS2, lys2, ura3, leu2::hisG, his3::hisG, trp1::hisG,*  *rpd3::HIS3MX, hos2:HIS3MX, TUP1-3V5::HIS3 MATalpha, ho::LYS2, lys2, ura3, leu2::hisG, his3::hisG, trp1::hisG,*  *rpd3::HIS3MX, hos2:HIS3MX, TUP1-3V5::HIS3* |
| FW3603 | *MATa, ho::LYS2, lys2, ura3, leu2::hisG, his3::hisG, trp1::hisG,*  *yap6::NatMX, TUP1-3V5::HIS3 MATalpha, ho::LYS2, lys2, ura3, leu2::hisG, his3::hisG, trp1::hisG,*  *yap6::NatMX, TUP1-3V5::HIS3* |
| FW3979 | *MATa, ho::LYS2, lys2, ura3, leu2::hisG, his3::hisG, trp1::hisG,*  *sok2::NatMX, TUP1-3V5::HIS3 MATalpha, ho::LYS2, lys2, ura3, leu2::hisG, his3::hisG, trp1::hisG,*  *sok2::NATMX, TUP1-3V5::HIS3* |
| FW3991 | *MATa, ho::LYS2, lys2, ura3, leu2::hisG, his3::hisG, trp1::hisG,*  *phd1::KanMX, TUP1-3V5::his3 MATalpha, ho::LYS2, lys2, ura3, leu2::hisG, his3::hisG, trp1::hisG,*  *phd1::KanMX, TUP1-3V5::his3* |
| FW4239 | *MATa, ho::LYS2, lys2, ura3, leu2::hisG, his3::hisG, trp1::hisG,*  *sok2::NatMX, yap6::NatMX, TUP1-3V5::HIS3 MATalpha, ho::LYS2, lys2, ura3, leu2::hisG, his3::hisG, trp1::hisG,*  *sok2::NatMX, yap6::NatMX, TUP1-3V5::HIS3* |
| FW4710 | *MATa, ho::LYS2, lys2, ura3, leu2::hisG, his3::hisG, trp1::hisG,*  *sok2::NatMX, phd1::KanMX, TUP1-3V5::HIS3 MATalpha, ho::LYS2, lys2, ura3, leu2::hisG, his3::hisG, trp1::hisG,*  *sok2::NatMX, phd1::KanMX, TUP1-3V5::HIS3* |
| FW4406 | *MATa, ho::LYS2, lys2, ura3, leu2::hisG, his3::hisG, trp1::hisG,*  *phd1::KanMX, yap6::NatMX, TUP1-3V5::HIS3 MATalpha, ho::LYS2, lys2, ura3, leu2::hisG, his3::hisG, trp1::hisG,*  *phd1::KanMX, yap6::NatMX, TUP1-3V5::HIS3* |
| FW4010 | *MATa, ho::LYS2, lys2, ura3, leu2::hisG, his3::hisG, trp1::hisG,*  *sok2::NATMX, phd1::KanMX, yap6::NATMX, TUP1-3V5::HIS3 MATalpha, ho::LYS2, lys2, ura3, leu2::hisG, his3::hisG, trp1::hisG,*  *sok2::NATMX, phd1::KanMX, yap6::NATMX, TUP1-3V5::HIS3* |
| FW5657 | *MATa, ho::LYS2, lys2, ura3, leu2::hisG, his3::hisG, trp1::hisG,*  *nrg1::KanMX, sok2::NatMX, phd1::KanMX, yap6::NatMX, TUP1-3V5::HIS3 MATalpha, ho::LYS2, lys2, ura3, leu2::hisG, his3::hisG, trp1::hisG,*  *nrg1::KanMX, sok2::NatMX, phd1::KanMX, yap6::NatMX, TUP1-3V5::HIS3* |
| FW7733 | *MATa, ho::LYS2, lys2, ura3, leu2::hisG, his3::hisG, trp1::hisG, pIME1-ime1::KanMX, trp1::pNH604-pIME1(spy)-sfGFP-IME1::TRP1, TUP1-3V5::HIS3 MATalpha, ho::LYS2, lys2, ura3, leu2::hisG, his3::hisG, trp1::hisG, pIME1-ime1::KanMX, trp1::pNH604-pIME1(spy)-sfGFP-IME1::TRP1, TUP1-3V5::HIS3* |
| FW8079 | *MATa, ho::LYS2, lys2, ura3, leu2::hisG, his3::hisG, trp1::hisG, pIME1-ime1::KanMX, trp1::pNH604-pIME1(WT)-sfGFP-IME1::TRP1, YAP6-3V5::KanMX MATalpha, ho::LYS2, lys2, ura3, leu2::hisG, his3::hisG, trp1::hisG, pIME1-ime1::KanMX, trp1::pNH604-pIME1(WT)-sfGFP-IME1::TRP1, YAP6-3V5::KanMX* |
| FW8085 | *MATa, ho::LYS2, lys2, ura3, leu2::hisG, his3::hisG, trp1::hisG, pIME1-ime1::KanMX, trp1::pNH604-pIME1(bs∆)-sfGFP-IME1::TRP1, YAP6-3V5::KanMX MATalpha, ho::LYS2, lys2, ura3, leu2::hisG, his3::hisG, trp1::hisG, pIME1-ime1::KanMX, trp1::pNH604-pIME1(bs∆)-sfGFP-IME1::TRP1, YAP6-3V5::KanMX* |
| FW8091 | *MATa, ho::LYS2, lys2, ura3, leu2::hisG, his3::hisG, trp1::hisG, pIME1-ime1::KanMX, trp1::pNH604-pIME1(spy)-sfGFP-IME1::TRP1, YAP6-3V5::KanMX MATalpha, ho::LYS2, lys2, ura3, leu2::hisG, his3::hisG, trp1::hisG, pIME1-ime1::KanMX, trp1::pNH604-pIME1(spy)-sfGFP-IME1::TRP1, YAP6-3V5::KanMX* |
| FW8081 | *MATa, ho::LYS2, lys2, ura3, leu2::hisG, his3::hisG, trp1::hisG, pIME1-ime1::KanMX, trp1::pNH604-pIME1(WT)-sfGFP-IME1::TRP1, SOK2-3V5::HIS3 MATalpha, ho::LYS2, lys2, ura3, leu2::hisG, his3::hisG, trp1::hisG, pIME1-ime1::KanMX, trp1::pNH604-pIME1(WT)-sfGFP-IME1::TRP1, SOK2-3V5::HIS3* |
| FW8087 | *MATa, ho::LYS2, lys2, ura3, leu2::hisG, his3::hisG, trp1::hisG, pIME1-ime1::KanMX, trp1::pNH604-pIME1(bs∆)-sfGFP-IME1::TRP1, SOK2-3V5::HIS3 MATalpha, ho::LYS2, lys2, ura3, leu2::hisG, his3::hisG, trp1::hisG, pIME1-ime1::KanMX, trp1::pNH604-pIME1(bs∆)-sfGFP-IME1::TRP1, SOK2-3V5::HIS3* |
| FW8093 | *MATa, ho::LYS2, lys2, ura3, leu2::hisG, his3::hisG, trp1::hisG, pIME1-ime1::KanMX, trp1::pNH604-pIME1(spy)-sfGFP-IME1::TRP1, SOK2-3V5::HIS3 MATalpha, ho::LYS2, lys2, ura3, leu2::hisG, his3::hisG, trp1::hisG, pIME1-ime1::KanMX, trp1::pNH604-pIME1(spy)-sfGFP-IME1::TRP1, SOK2-3V5::HIS3* |
| FW8083 | *MATa, ho::LYS2, lys2, ura3, leu2::hisG, his3::hisG, trp1::hisG, pIME1-ime1::KanMX, trp1::pNH604-pIME1(WT)-sfGFP-IME1::TRP1, PHD1-3V5::KanMX MATalpha, ho::LYS2, lys2, ura3, leu2::hisG, his3::hisG, trp1::hisG, pIME1-ime1::KanMX, trp1::pNH604-pIME1(WT)-sfGFP-IME1::TRP1, PHD1-3V5::KanMX* |
| FW8089 | *MATa, ho::LYS2, lys2, ura3, leu2::hisG, his3::hisG, trp1::hisG, pIME1-ime1::KanMX, trp1::pNH604-pIME1(bs∆)-sfGFP-IME1::TRP1, PHD1-3V5::KanMX MATalpha, ho::LYS2, lys2, ura3, leu2::hisG, his3::hisG, trp1::hisG, pIME1-ime1::KanMX, trp1::pNH604-pIME1(bs∆)-sfGFP-IME1::TRP1, PHD1-3V5::KanMX* |
| FW8095 | *MATa, ho::LYS2, lys2, ura3, leu2::hisG, his3::hisG, trp1::hisG, pIME1-ime1::KanMX, trp1::pNH604-pIME1(spy)-sfGFP-IME1::TRP1, PHD1-3V5::KanMX MATalpha, ho::LYS2, lys2, ura3, leu2::hisG, his3::hisG, trp1::hisG, pIME1-ime1::KanMX, trp1::pNH604-pIME1(spy)-sfGFP-IME1::TRP1, PHD1-3V5::KanMX* |
| FW7650 | *MATa, ho::LYS2, lys2, ura3, leu2::hisG, his3::hisG, trp1::hisG, sok2::NatMX, yap6::NatMX, phd1::KanMX, pIME1-ime1::KanMX, trp1::pNH604-pIME1(WT)-sfGFP-IME1::TRP1, TUP1-3V5::HIS3 MATalpha, ho::LYS2, lys2, ura3, leu2::hisG, his3::hisG, trp1::hisG, sok2::NatMX, yap6::NatMX, phd1::KanMX, pIME1-ime1::KanMX, trp1::pNH604-pIME1(WT)-sfGFP-IME1::TRP1, TUP1-3V5::HIS3* |
| FW8420 | *MATa, ho::LYS2, lys2, ura3, leu2::hisG, his3::hisG, trp1::hisG, sok2::NatMX, yap6::NatMX, phd1::KanMX, pIME1-ime1::KanMX, trp1::pNH604-pIME1(bs∆)-sfGFP-IME1::TRP1, TUP1-3V5::HIS3 MATalpha, ho::LYS2, lys2, ura3, leu2::hisG, his3::hisG, trp1::hisG, sok2::NatMX, yap6::NatMX, phd1::KanMX, pIME1-ime1::KanMX, trp1::pNH604-pIME1(bs∆)-sfGFP-IME1::TRP1, TUP1-3V5::HIS3* |
| FW8177 | *MATa, ho::LYS2, lys2, ura3, leu2::hisG, his3::hisG, trp1::hisG, yap6::NatMX, sok2::NatMX, phd1::KanMX, pIME1-ime1::KanMX, trp1::pNH604-pIME1(spy)-sfGFP-IME1::TRP1, TUP1-3V5::HIS3 MATalpha, ho::LYS2, lys2, ura3, leu2::hisG, his3::hisG, trp1::hisG, yap6::NatMX, sok2::NatMX, phd1::KanMX, pIME1-ime1::KanMX, trp1::pNH604-pIME1(spy)-sfGFP-IME1::TRP1, TUP1-3V5::HIS3* |
| FW5199 | *MATa, ho::LYS2, lys2, ura3, leu2::hisG, his3::hisG, trp1::hisG,*  *pRS306-pCTS1-2xmCherry-SV40NLS MATalpha, ho::LYS2, lys2, ura3, leu2::hisG, his3::hisG, trp1::hisG,*  *pRS306-pCTS1-2xmCherry-SV40NLS* |
| FW7473 | *MATa, ho::LYS2, lys2, ura3, leu2::hisG, his3::hisG, trp1::hisG,*  *pRS306-pCTS1-2xmCherry-SV40NLS, YAP6-mNeongreen(Yeast Optimized)::NatMX MATalpha, ho::LYS2, lys2, ura3, leu2::hisG, his3::hisG, trp1::hisG,*  *pRS306-pCTS1-2xmCherry-SV40NLS, YAP6-mNeongreen(Yeast Optimized)::NatMX* |
| FW7475 | *MATa, ho::LYS2, lys2, ura3, leu2::hisG, his3::hisG, trp1::hisG,*  *pRS306-pCTS1-2xmCherry-SV40NLS, SOK2-mNeongreen(Yeast Optimized)::NatMX MATalpha, ho::LYS2, lys2, ura3, leu2::hisG, his3::hisG, trp1::hisG,*  *pRS306-pCTS1-2xmCherry-SV40NLS, SOK2-mNeongreen(Yeast Optimized)::NatMX* |
| FW7477 | *MATa, ho::LYS2, lys2, ura3, leu2::hisG, his3::hisG, trp1::hisG,*  *pRS306-pCTS1-2xmCherry-SV40NLS, PHD1-mNeongreen(Yeast Optimized)::NatMX MATalpha, ho::LYS2, lys2, ura3, leu2::hisG, his3::hisG, trp1::hisG,*  *pRS306-pCTS1-2xmCherry-SV40NLS, PHD1-mNeongreen(Yeast Optimized)::NatMX* |
| FW7644 | *MATa, ho::LYS2, lys2, ura3, leu2::hisG, his3::hisG, trp1::hisG,*  *pRS306-pCTS1-2xmCherry-SV40NLS, TUP1-mNeongreen(Yeast Optimized)::NatMX MATalpha, ho::LYS2, lys2, ura3, leu2::hisG, his3::hisG, trp1::hisG,*  *pRS306-pCTS1-2xmCherry-SV40NLS, TUP1-mNeongreen(Yeast Optimized)::NatMX* |
| FW7642 | *MATa, ho::LYS2, lys2, ura3, leu2::hisG, his3::hisG, trp1::hisG,*  *pRS306-pCTS1-2xmCherry-SV40NLS, CYC8-mNeongreen(Yeast Optimized)::NatMX MATalpha, ho::LYS2, lys2, ura3, leu2::hisG, his3::hisG, trp1::hisG,*  *pRS306-pCTS1-2xmCherry-SV40NLS, CYC8-mNeongreen(Yeast Optimized)::NatMX* |
