# Supplementary material for: Regulated repression governs the cell fate promoter controlling yeast meiosis
Source: Nat Commun. 2020 May 8;11:2271. doi: 10.1038/s41467-020-16107-w (PMC7210989; doi:10.1038/s41467-020-16107-w)
Supplement: Supplementary file 5 — Reporting Summary [file 41467_2020_16107_MOESM5_ESM.pdf]

nature research

Corresponding author(s): Folkert van Werven

Last updated by author(s): Mar 21, 2020

Reporting Summary

Nature Research wishes to improve the reproducibility of the work that we publish. This form provides structure for consistency and transparency in reporting. For further information on Nature Research policies, see Authors & Referees and the Editorial Policy Checklist.

Statistics

For all statistical analyses, confirm that the following items are present in the figure legend, table legend, main text, or Methods section.

n/a	Confirmed	
☐	☒	The exact sample size (*n*) for each experimental group/condition, given as a discrete number and unit of measurement
☒	☐	A statement on whether measurements were taken from distinct samples or whether the same sample was measured repeatedly
☐	☒	The statistical test(s) used AND whether they are one- or two-sided<br>*Only common tests should be described solely by name; describe more complex techniques in the Methods section.*
☒	☐	A description of all covariates tested
☒	☐	A description of any assumptions or corrections, such as tests of normality and adjustment for multiple comparisons
☐	☒	A full description of the statistical parameters including central tendency (e.g. means) or other basic estimates (e.g. regression coefficient) AND variation (e.g. standard deviation) or associated estimates of uncertainty (e.g. confidence intervals)
☐	☒	For null hypothesis testing, the test statistic (e.g. *F*, *t*, *r*) with confidence intervals, effect sizes, degrees of freedom and *P* value noted<br>*Give P values as exact values whenever suitable.*
☒	☐	For Bayesian analysis, information on the choice of priors and Markov chain Monte Carlo settings
☒	☐	For hierarchical and complex designs, identification of the appropriate level for tests and full reporting of outcomes
☒	☐	Estimates of effect sizes (e.g. Cohen's *d*, Pearson's *r*), indicating how they were calculated

Our web collection on statistics for biologists contains articles on many of the points above.

Software and code

Policy information about availability of computer code

Data collection	NIS-elements (Nikon); Applied Biosystems 7500 Fast Real-Time PCR System (Thermo Fisher Scientific)
Data analysis	YeTFaSCo database version 1.02 (http://yetfasco.ccbr.utoronto.ca); GraphPad Prism 8.2.0 (www.graphpad.com); ImageJ 1.52a (http://imagej.nih.gov/ij); StarSearch (https://www.seas.upenn.edu/~rajlab/StarSearch/launch.html)

For manuscripts utilizing custom algorithms or software that are central to the research but not yet described in published literature, software must be made available to editors/reviewers. We strongly encourage code deposition in a community repository (e.g. GitHub). See the Nature Research guidelines for submitting code & software for further information.

Data

Policy information about availability of data

All manuscripts must include a data availability statement. This statement should provide the following information, where applicable:

- Accession codes, unique identifiers, or web links for publicly available datasets
- A list of figures that have associated raw data
- A description of any restrictions on data availability

No public available datasets are involved.
Raw data: Fig. 1b-i, Fig. 2b-c, Fig. 3b-d, Fig. 4a-c, Fig. 5a-e, Fig. 6a-c, Fig. 7a-c, Fig. 8b-d, Suppl. Fig. 1, Suppl. Fig. 2, Suppl. Fig. 4a, Suppl. Fig. 5a-b, Suppl. Fig. 6, Suppl. Fig. 7, Suppl. Fig. 8.
There is no restriction on data availability.

Field-specific reporting

Please select the one below that is the best fit for your research. If you are not sure, read the appropriate sections before making your selection.

☒ Life sciences ☐ Behavioural & social sciences ☐ Ecological, evolutionary & environmental sciences

For a reference copy of the document with all sections, see nature.com/documents/nr-reporting-summary-flat.pdf

Life sciences study design

All studies must disclose on these points even when the disclosure is negative.

Sample size	Sample sizes are indicated in the legends, methods, and in the figures themselves.
Data exclusions	No data was excluded from the analyses.
Replication	In general, two (mostly three) or more replicates were performed for each experiment. For the single molecule RNA-FISH experiment, the number of cells used for the analyses are indicated. For some experiments, representative experiments are displayed. In particular, meiosis time courses (DAPI based assay) can be variable between repeats, but for every repeat all the controls were included and trends observed were comparable. For the western blots, representative experiments are also shown.
Randomization	Not applicable
Blinding	Blinding was not possible nor necessary as we used well-defined quantitative techniques for all the measurements made.

Reporting for specific materials, systems and methods

We require information from authors about some types of materials, experimental systems and methods used in many studies. Here, indicate whether each material, system or method listed is relevant to your study. If you are not sure if a list item applies to your research, read the appropriate section before selecting a response.

Materials & experimental systems

n/a	Involved in the study	
☐	☒	Antibodies
☒	☐	Eukaryotic cell lines
☒	☐	Palaeontology
☒	☐	Animals and other organisms
☒	☐	Human research participants
☒	☐	Clinical data

Methods

n/a	Involved in the study	
☒	☐	ChIP-seq
☒	☐	Flow cytometry
☒	☐	MRI-based neuroimaging

Antibodies

Antibodies used

Anti-V5 agarose beads (Sigma-Aldrich, cat. no. A7345-1ML, monocloncal antibody produced in mouse, clone V5-10);
anti-H3 antibody (Abcam, cat. no. ab1791, polyclonal antibody produced in rabbit);
anti-V5 antibody (Invitrogen, cat. no. R960-25, monoclonal antibody produced in mouse);
anti-hexokinase antibody (Stratech, cat. no. H2035, antibody produced in rabbit);
HRP-conjugated anti-mouse antibody (GE Healthcare, cat. no. NA931-1ML, antibody produced in sheep);
HRP-conjugated anti-rabbit antibody (GE Healthcare, cat. no. NA934-1ML, antibody produced in donkey);
IRDye 800CW (LI-COR, cat. no. 926-32210, anti-mouse IgG secondary antibody produced in goat);
IRDye 680RD (LI-COR, cat. no. 926-68071, anti-rabbit IgG secondary antibody produced in goat)

Validation

Anti-V5 agarose beads (Sigma-Aldrich) were used to immunoprecipitate proteins fused with V5 epitope in chromatin immunoprecipitation (ChIP) experiments in this manuscript. The product information datasheet released by the manufacturer indicates that the product recognizes recombinant proteins tagged with V5 in transfected mammalian cells, and is therefore suitable for the immunoprecipitation of V5-tagged proteins from cell lysates. This product has also been used in more than 70 peer-reviewed publications, according to the manufacturer's website.

Anti-H3 antibodies (Abcam) were coupled to Dynabeads Protein A to immunoprecipitate histone H3 in ChIP experiments in this manuscript. The antibody was raised against a synthetic peptide containing a portion of human histone H3 fused to keyhole limpet haemocyanin. According to the manufacturer's website, the antibody recognizes a 15kDa protein on western blot and can be blocked by H3 peptide. The use of this product has also been validated in multiple applications including ChIP.

For Western blotting, anti-V5 (Invitrogen) and anti-hexokinase (Stratech) antibodies were used as primary antibodies. The use of the anti-V5 antibody was analysed by the manufacturer using immunofluorescence analysis and western blotting. According to the manufacturer's website, the anti-V5 antibody has been included in at least 40 published figures. The specificity of this

product was also validated in this manuscript by western blotting in Fig. 1e, 1g, and Suppl. Fig. 4a. The anti-hexokinase antibody was purchased from a company that performs thorough quality and validation checks. This use of this product has been described in multiple publications and its specificity was also validated in this manuscript by western blotting in Fig. 1e and 1g.
